# A Comprehensive and Historical Review of Minimally Invasive Scoliosis Surgery in Adolescent Idiopathic Scoliosis: An Analysis of Research Trends and Hotspots

**DOI:** 10.3390/jcm14165676

**Published:** 2025-08-11

**Authors:** Hong Jin Kim, Dong Yun Kim, Jae Hyuk Yang, Jungwook Lim, Seung Woo Suh

**Affiliations:** 1 Department of Orthopaedic Surgery, Kyung-in Regional Military Manpower Administration, Suwon 16440, Republic of Korea; hongjin0925@naver.com; 2 Department of Medicine, Korea University College of Medicine, Seoul 02841, Republic of Korea; okkdy0628@korea.ac.kr; 3 Department of Orthopaedic Surgery, Korea University Anam Hospital, Seoul 02841, Republic of Korea; kuspine@korea.ac.kr; 4 Department of Orthopaedic Surgery, Korea University Guro Hospital, Seoul 08308, Republic of Korea; jlim2012@gmail.com

**Keywords:** adolescent idiopathic scoliosis, minimally invasive scoliosis surgery, vertebral body tethering, bibliometric analysis, research trends

## Abstract

Over the past two decades, interest in minimally invasive scoliosis surgery (MISS) for adolescent idiopathic scoliosis (AIS) has grown substantially, driven by advancements in growth-based surgical techniques. Given the substantial advancements in MISS for AIS, investigating the bibliometric data of the scientific literature is crucial to understanding the current research trend and providing valuable insights into its future directions. However, limited information on MISS for AIS exists in the literature. The publication data related to MISS for AIS from 2004 to 2024 were exported from the Web of Science. The research output between 2004 and 2024 was 373 for publication volume, 7760 for citations, and 46 for h-index. The annual publication and citation trend over time showed a gradual increase with fluctuations up until 2017, followed by a sharp upward trend starting in 2018. The foremost countries and affiliations in this field were the United States and Montreal University in Canada, respectively. The top 10 most-cited articles on MISS in AIS predominantly focused on the topic of vertebral body tethering (VBT). Among the productive authors, most contributions were focused on VBT, while several authors in South Korea significantly contributed to the study of MISS via a posterior approach. Historical development of VBT and posterior MISS identified their current advantages and limitations and highlighted potential future research directions.

## 1. Introduction

Posterior spinal fusion, comprising pedicle screw instrumentation (PSI), and correctional maneuvers, such as rod derotation (RD) and posterior fusion, is required for severe cases of adolescent idiopathic scoliosis (AIS) to restore coronal and sagittal alignment [1]. In general, surgical treatment was performed in the patients with AIS who were at risk of curve progression in adulthood, typically defined as a Cobb angle greater than 40–45° in the thoracolumbar region or greater than 50° in the thoracic region [1,2]. While this traditional surgical indication based on curve magnitude remains valid, smaller curves—particularly thoracolumbar and lumbar curves—can also result in clinical deformities, such as coronal imbalance and truncal shift, leading to dissatisfaction and the need for surgical intervention [3]. Although the primary goal of surgical treatment is to prevent scoliosis progression and restore spinal alignment by achieving a spinal fusion, cosmetic concerns—such as rib hump deformity—should also be considered in the decision-making process. Conventional open scoliosis surgery (COSS) has the drawback of potential cosmetic dissatisfaction due to the long midline incision on the back [4]. However, minimally invasive scoliosis surgery (MISS) for AIS was considered technically challenging, given the need to correct larger curve magnitudes, address longer instrumentation levels and axially rotated vertebrae, and operate within limited surgical spaces for specific correctional maneuvers [5,6].

Substantial interest in MISS for AIS has grown, driven by advancements in growth-based surgical techniques [7]. In particular, vertebral body tethering (VBT), a compression-based growth modulation via the anterior approach, has been increasingly adopted worldwide for deformity correction in skeletally immature AIS patients, offering the advantage of motion preservation [8,9,10]. Concurrently, MISS via the posterior approach—utilizing PSI, correctional maneuvers, and posterior fusion through two or three small incisions, each approximately two inches—has been introduced to enhance cosmetic outcomes, minimize surgical morbidity, and achieve radiological outcomes comparable to COSS [4,11].

Over the past two decades, rapidly emerging in-depth research has made it challenging to identify trends and hotspots in the field of MISS for AIS. Given the substantial advancements in MISS for AIS, it is crucial to understanding the current research trend and providing valuable insights into its future directions. Therefore, in this review article with a bibliometric search, we aim to explore the historical development of MISS in AIS, to understand current research trends, and to discuss emerging hotspots and future directions.

## 2. Materials and Methods

**Figure 1:**
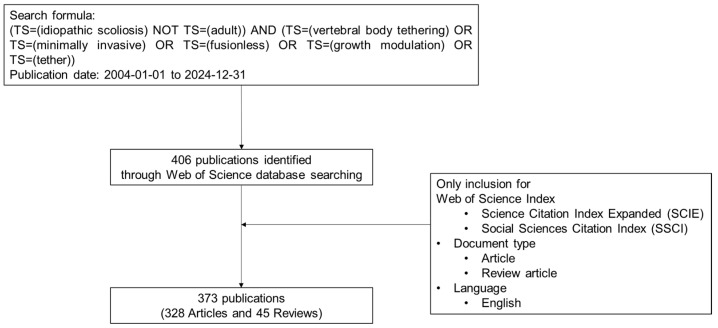
Search strategy.

We seek to address the following research questions for this comprehensive and historical review: What are the research trends and hotspots in MISS of AIS between 2004 and 2024? The Web of Science (WOS) database was utilized to search for relevant published articles. To focus on the subject of MISS in AIS, the following search formula was applied: (TS = (idiopathic scoliosis) NOT TS = (adult)) AND (TS = (vertebral body tethering) OR TS = (minimally invasive) OR TS = (fusionless) OR TS = (growth modulation) OR TS = (tether)) with a publication date range from 1 January 2004 to 31 December 2024. This search initially identified 406 published articles. Articles were excluded if they did not meet specific criteria, including the WOS index (not indexed in the Science Citation Index Expanded or the Social Sciences Citation Index), document type (not original articles or reviews), or language (not published in English). Finally, 373 relevant published papers were included in this bibliometric analysis (Figure 1).

Two independent researchers participated in data collection, extraction, and cross-checking. A descriptive statistical analysis was performed manually and visualized using bar charts created in Microsoft Excel. To analyze the text data exported from the WOS database, bibliometric software such as VOSviewer 1.6.15 (Leiden University, Leiden, The Netherlands) was employed. Links between nodes indicated co-authorship or co-citation relationships, while the total link strength (TLS) acted as a weight attribute, reflecting the overall strength of connections associated with each node.

## 3. Results

### 3.1. Research Outputs

**Figure 2:**
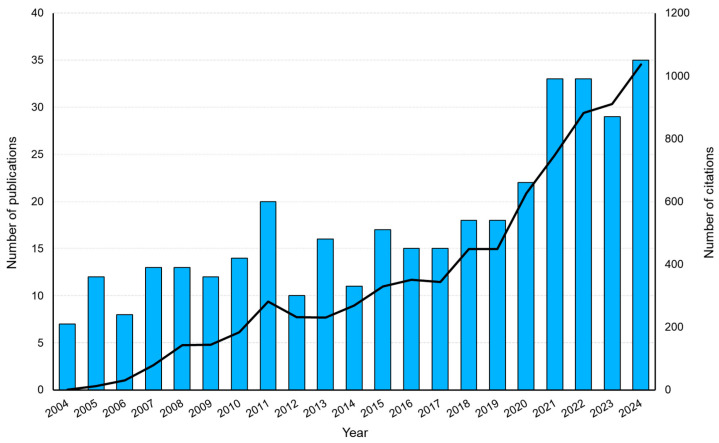
Annual scientific publications and citations for minimally invasive scoliosis surgery of adolescent idiopathic scoliosis.

**Figure 3:**
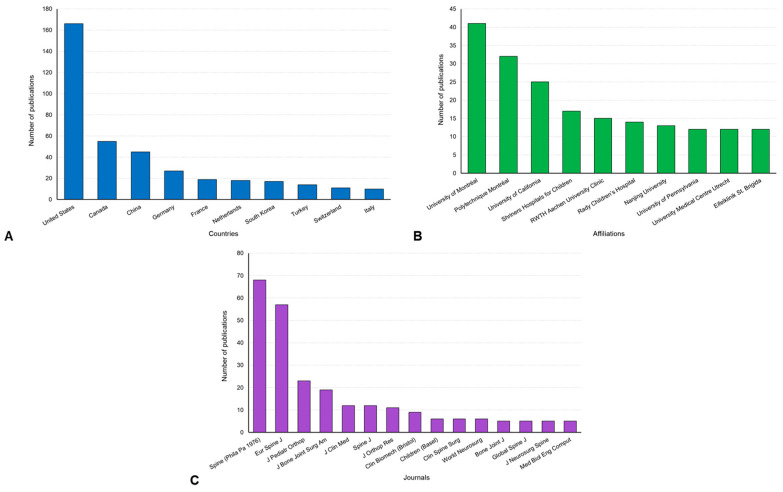
Publication distributions based on countries (**A**), affiliations (**B**), and journals (**C**) for minimally invasive scoliosis surgery of adolescent idiopathic scoliosis.

The publication volume was 373 from 2004 to 2024, comprising 328 articles and 45 reviews. Citation volume was 7760 in total and 5780 without self-citation. The h-index for MISS of AIS was 46 (Table 1). The annual publication trend over time showed a gradual increase with fluctuations up until 2017, followed by a sharp upward trend starting in 2018. Similarly, the annual citation trend over time steadily grew until 2017, after which it exhibited a significant increase beginning in 2018 (Figure 2). The United States is the leading country in terms of publications on MISS in AIS (Figure 3A). Among institutions, the University of Montreal and Polytechnique Montreal in Canada made the most substantial contributions (Figure 3B). The primary journals publishing on the topic of MISS in AIS include the Spine (Phila Pa 1976), and the European Spine Journal (Eur Spine J) (Figure 3C).

### 3.2. Most Influential Papers and Authors

Based on citation volumes, we identified the top 10 most-cited original articles on MISS in AIS, which predominantly focus on the topic of VBT. These papers were published in the four issues of the Spine (Phila Pa 1976), four issues of the J Bone Joint Surg Am, and two issues of the Eur Spine J. The most influential paper, authored by Samdani et al., reported the two-year outcomes of anterior VBT for idiopathic scoliosis, entitled “Anterior vertebral body tethering for idio-pathic scoliosis: two-year results”. This retrospective study demonstrated that anterior VBT is a safe procedure capable of achieving progressive correction [12]. Notably, the first paper on VBT (Growth Modulation by Means of Anterior Tethering Resulting in Progressive Correction of Juvenile Idiopathic Scoliosis A Case Report) ranks fourth on the list, with 119 citations, averaging 7.44 citations per year [13] (Table 2).

Among the most productive authors in the field of MISS for AIS, Parent S ranked first, with the highest number of publications (32), total citations (647), and an h-index of 15. Most productive authors were located in North America (USA and Canada). Baroncini A (6th rank) and Trobisch PD (7th rank) in Germany were the most productive authors in Europe. Among the productive authors, most contributions were focused on VBT, while several authors in South Korea were also recorded: Chang DG, Kim HJ, Suh SW, and Yang JH who studied MISS via a posterior approach (Table 3).

### 3.3. Co-Authorship

**Figure 4:**
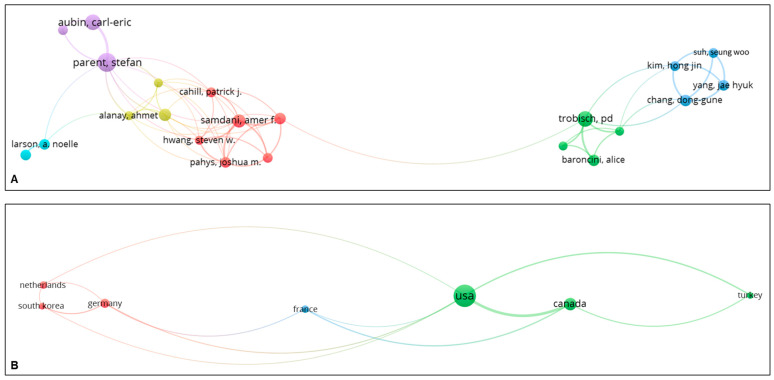
Co-authorship analysis with network map based on authors (**A**) and countries (**B**).

Among authors with a minimum of seven publications, Samdani AF had the highest TLS of 39, followed by Parent S (TLS 35), Pahys JM (TLS 29), and Trobisch PD (TLS 29). Of the six identified clusters, five were associated with VBT, while only one cluster (blue) focused on MISS via the posterior approach (Figure 4A). Among countries with a minimum of 10 publications, the United States had the highest TLS of 32, followed by Canada (TLS, 25). The United States, in particular, had well-established networks with Canada and European countries such as the Netherlands and Germany (Figure 4B).

**Figure 5:**
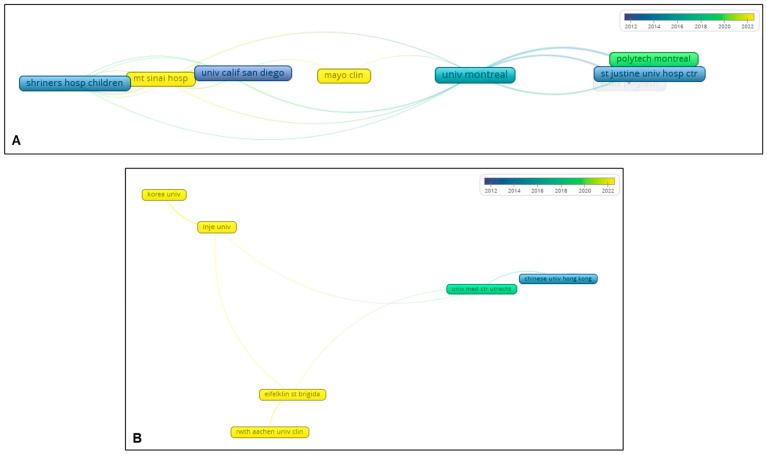
Co-authorship analysis with overlay visualization based on affiliations, comprising two independent clusters (**A**,**B**).

Based on affiliations with a minimum of seven publications, overlay visualization revealed two distinct clusters. The first cluster was associated with studies on VBT conducted in the United States and Canada (Figure 5A). The second cluster included affiliations from Europe and Asia, focusing on MISS via the posterior approach, particularly in South Korea, as well as on VBT (Figure 5B).

### 3.4. Co-Citation Analysis

**Figure 6:**
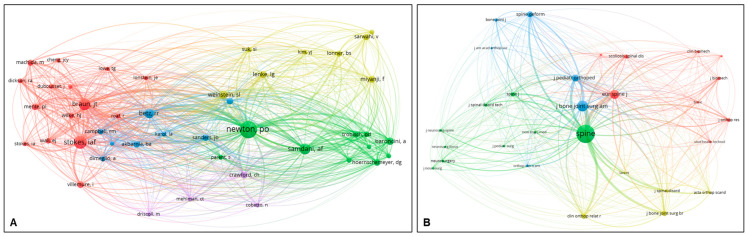
Co-citation analysis by authors (**A**) and journals (**B**).

In the co-citation analysis of authors with at least 30 citations, Newton PO had the highest TLS of 5202, followed by Braun JT (2667) and Stokes IAF (2524). Among the four identified clusters, most were associated with research on VBT that was mainly conducted in North America (USA and Canada); however, the yellow cluster was linked to PSI by posterior approach (Figure 6A). For the journals with at least 50 citations, the Spine (Phila Pa 1976) had the highest TLS of 71,591, followed by J Bone Joint Surg Am (34,237), and the Eur Spine J (24,669) (Figure 6B).

### 3.5. Co-Occurrence Analysis of Keywords

**Figure 7:**
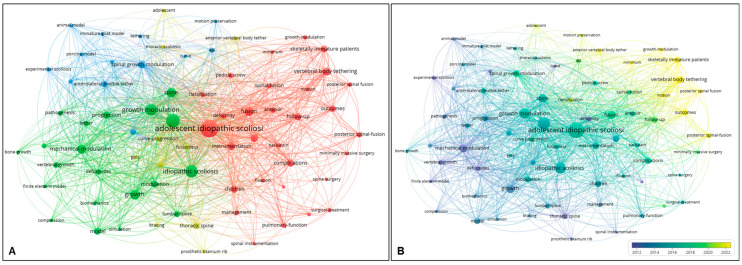
Co-occurrence analysis with overlay visualization based on keywords (**A**,**B**).

The co-occurrence analysis identified key contributing keywords for MISS in AIS, including growth modulation (TLS, 457), VBT (TLS, 219), instrumentation (TLS, 205), and complications (TLS, 173). The network map revealed four clusters: the blue cluster represented preclinical studies on VBT, the green cluster focused on biomechanical studies of VBT, the yellow cluster addressed VBT in thoracic scoliosis, and the red cluster was linked to clinical studies of MISS. Within the red cluster, minimally invasive surgery was connected to posterior spinal fusion and instrumentation, indicating a focus on MISS via the posterior approach. VBT, on the other hand, was associated with follow-up, outcomes, and posterior spinal fusion, highlighting topics related to surgical outcomes based on follow-up duration, hybrid surgical approaches (VBT and posterior spinal fusion), and complications (Figure 7A). Over the past two decades, VBT has progressively advanced from experimental research to biomechanical studies and, more recently, to clinical applications in the overlay visualization of co-occurrence analysis. The current focus of MISS research is clearly on VBT and its associated complications. Notably, despite being a fusionless technique, ‘posterior spinal fusion’ also emerged as a recent keyword in MISS for AIS (Figure 7B).

## 4. Discussion

### 4.1. Research Trends from Past to Present to Future

For the topic of MISS in AIS between 2004 and 2024, our bibliometric study revealed a dramatic increase in interest in MISS since 2017. The global publication trend predominantly stemmed from research on VBT, which has evolved progressively from basic studies to clinical applications. It is evident that VBT is a research hotspot in MISS for AIS, with current studies focusing on complications such as tether breakage, efforts to address these issues, and the expansion of surgical indications based on scoliotic curve types [22]. Meanwhile, MISS via the posterior approach, which evolved from COSS, could be observed in the yellow cluster of co-citation analysis and blue cluster of co-authorship analysis. Interestingly, while VBT and MISS via posterior approach fall under the same category of MISS, their characteristics differ significantly. These distinctive characteristics offer valuable insights into how future research trends in MISS might ideally evolve, considering the current hotspots of hybrid surgery in VBT.

### 4.2. Global Difference on MISS Trend

From a regional perspective, the United States leads the field of MISS research in AIS, with the highest publication volume. The first case of anterior VBT was reported in 2010 by Crawford CH and Lenke LG, both affiliated with institutions in the United States [13]. In Canada, the University of Montreal and Polytechnique Montreal have been actively involved in biomechanical and clinical studies of VBT [23]. With the safety and effectiveness of VBT established, MISS via the anterior approach (i.e., VBT) has gained global popularity, particularly in North America and Europe, with notable contributions from Germany [24]. In Asia, China ranks third in publication volume, contributing widely to MISS research, including a meta-analysis [25]. South Korea ranks seventh in publication volume and is distinguished by its focus on MISS via the posterior approach, rather than VBT as highlighted in this bibliometric information.

The co-citation analysis revealed that Newton PO was the most influential author, closely connected with Samdani AF. Together, they contributed six of the top ten most-cited original articles on MISS in AIS, focusing on the clinical outcomes and experimental models of VBT. For biomechanical growth modulation studies, Stokes IAF and Braun JT were among the most influential authors, contributing two of the top ten most-cited original articles on MISS in AIS. In the co-citation network map, a cluster (yellow) associated with PSI-related research was identified, which appeared to be linked to MISS via the posterior approach. This cluster included prominent surgeons in the USA for their work on AIS, PSI, and associated techniques such as the free-hand technique [26]. Moreover, Sarwahi V, affiliated with Montefiore Medical Center, was the first to describe the MISS technique for AIS. The initial posterior MISS approach was characterized by three key features: three 4-cm midline incisions, fusion of all facet joints, and skip pedicle screw fixation using the free-hand technique [27]. However, considering the relatively low representation of influential papers in MISS, this approach does not seem to be as popular. However, as efforts to address complications associated with VBT and to expand its indications are ongoing, as highlighted in the keyword analysis, integrating a hybrid approach with the posterior technique may become necessary [28].

### 4.3. VBT

Based on the keyword co-occurrence analysis, fusionless and growth modulation in AIS are evidently research hotspots of MISS. Historically, the potential for surgery like VBT has been recognized since temporary epiphysiodesis in the knee was established as a successful procedure for correcting knee deformities [29]. This technique involves modulating growth by inhibiting physical growth on a hemi-side under the Hueter–Volkmann principle, allowing continued growth on the other [30]. In VBT, compression force is applied to the convex side, hindering the growth of vertebral endplates through spinal implants [31]. As highlighted in our keyword analysis, this technique was initially investigated through animal and biomechanical studies, demonstrating its potential for curve correction while preserving disk integrity and spinal motion. Building on these foundational studies, clinical applications have proven successful, and VBT now appears to be well-established as a fusionless surgical approach [24].

VBT is based on the hypothesis of curve progression driven by asymmetrical spinal growth. Relative anterior spinal overgrowth, resulting from a disproportion of endochondral–membranous bone growth, leads to thoracic hypokyphosis and is associated with rotational instability [32,33]. VBT aims to reverse these pathological forces by applying compression to the convex side of the scoliotic curve, thereby off-loading the concave endplates and stimulating differential vertebral growth [9]. This modulation of growth facilitates gradual correction of the scoliotic deformity and alters the mechanical environment of the physis of vertebra [9,34]. A qualitative study suggested that the preoperative expectations of AIS patients vary widely, including goals such as pain relief, improved or preserved physical function, long-term stability, and improved emotional well-being [35]. In comparative studies between VBT and posterior spinal fusion, patients who underwent VBT experienced significantly less motion loss at 2-year follow-up [36]. However, a non-randomized clinical trial found that posterior spinal fusion was associated with a higher rate of favorable postoperative outcomes, greater correction in both the main thoracic and compensatory lumbar curves, and fewer complications compared to VBT [37]. Therefore, in light of the diverse expectations of AIS patients, surgeons should carefully consider individual goals and clinical factors when selecting the most appropriate surgical approach.

Considering the current findings, VBT appears to be recognized as the most promising technique in the surgical treatment of AIS [7,10]. However, recent challenges in VBT, such as tether breakage, revision rate, and its limitations in indications due to basically convex-side implantation, have led to an emerging trend of hybrid surgeries [22,28,38]. Future hotspots on VBT are anticipated to focus on reducing complications and expanding its indications to address a broader range of scoliotic types [39,40].

### 4.4. MISS by Posterior Approach

VBT remains the cornerstone of MISS in AIS, while MISS via the posterior approach—referred to as the coin-hole technique—is more frequently reported in South Korea [4,8]. MISS using posterior approaches uses two or three shorter incisions to address cosmetic issue in the back and facetal fusion, which are involved in less extensive soft tissue dissection and have a decreased area for subperiosteal exposures. Since this technique is based on the posterior skin redundancy, preliminary studies has been reported as wound complication and infection [5,41]. Sarwahi et al. emphasized tissue preservation should be prioritized over small-sized incision, developing a single long-incision minimally invasive surgery [42]. Meanwhile, in South Korea, MISS by posterior approach is widely conducted by Suh et al. They utilized a tubular retractor system and specialized reamer to reduce skin and soft tissue damage [43]. Current data in both groups (Sarwahi V’s technique and coin-hole technique) suggested that MISS by posterior approach provided equivalent radiological and clinical outcomes with a superior results of estimated blood loss and hospitalization in patients with AIS up to a moderate-to-severe degree of curve magnitude [4]. MISS by posterior approach aims to reduce wound size and preserve soft tissue compared to conventional surgery. Therefore, the surgical applicability demonstrated in previous studies on open surgical approaches may be extended to MISS as well. However, according to the bibliometric analysis, MISS constitutes only a minor portion of the current data (South Korea ranked 7th in MISS of AIS). From the perspective of future research hotspots, considering the need to address complications associated with VBT and to adapt VBT to various curve types, a combined approach integrating MISS may emerge as a novel surgical technique in the future.

Compared to VBT, the coin-hole technique has drawbacks, such as the loss of motion segments and its related complications, as well as a limited working field [44]. However, since it is developed based on COSS, it offers the advantages of well-established fusion level determination and applicability to all types of scoliotic curves [43]. Both techniques share cosmetic advantages as MISS procedures, with VBT involving an incision along the right mid-axillary line and the coin-hole technique utilizing incisions along the midline of the back. Therefore, our analysis not only provides a comprehensive understanding of the developmental processes and regional characteristics of these techniques but also offers valuable insights for suggesting future directions for their advancement.

### 4.5. Limitations

Our bibliometric analysis had several limitations. First, the study relied on a specific search formula, and because the retrieved data are sensitive to the search criteria, some relevant articles may have been excluded [45]. Nonetheless, we aimed to design a balanced search formula to comprehensively capture articles related to MISS in AIS. Second, the data were collected exclusively from the WOS database, which inevitably excluded some relevant articles available in other databases, such as Scopus, Embase, and PubMed, due to differences in export formats. Despite these limitations, our analysis effectively identified research trends in MISS and provided valuable insights into forecasting future developmental directions by comparing global and domestic research trends.

## 5. Conclusions

Over the past decades, there has been substantial and rapidly growing interest in MISS for AIS, with a sharp upward trend in publications and citations since 2018. The field is primarily driven by advancements in growth-based surgical techniques with the highest publications in North America. VBT—a compression-based growth modulation and fusionless technique—is identified as the foremost research hotpot, having deeply progressed from experimental and biomechanical studies to successful clinical applications that preserve flexibility. Recent research on VBT focuses on reducing complications like tether breakage and expanding surgical indications based on curve types. Concurrently, posterior-based MISS, as a fusion-based technique with small-sized incisions, serves as an alternative approach to MISS for AIS, offering cosmetic advantages and applicability to all scoliotic curve types. Although it is widely practiced in South Korea, which ranks seventh in publication volume, it still represents a relatively minor portion of the global literature. Therefore, our analysis not only provides a comprehensive understanding of the developmental processes and regional characteristics of these techniques but also offers valuable insights for suggesting future directions for their advancement.

## Figures and Tables

**Table 1:** A 21-year global and national publication volume of minimally invasive scoliosis surgery in adolescent idiopathic scoliosis.

Outputs	Publications	Citing Articles	Citations	H-Index
Research	373	3924	7760	46
Specific	Article: 328 Review: 45	Without self-citations: 3628	Without self-citations: 5780 Average per item: 20.8	

**Table 2:** Top 10 cited original articles for minimally invasive scoliosis surgery of adolescent idiopathic scoliosis.

Rank	Author	Year	Title	Journal	Citations	
					Total	Average per Year
1	Samdani AF, et al. [12]	2014	Anterior vertebral body tethering for idiopathic scoliosis: two-year results	Spine (Phila Pa 1976)	153	12.75
2	Samdani AF, et al. [14]	2015	Anterior vertebral body tethering for immature adolescent idiopathic scoliosis: one-year results on the first 32 patients	Eur Spine J	127	11.55
3	Newton PO, et al. [15]	2018	Anterior Spinal Growth Tethering for Skeletally Immature Patients with Scoliosis A Retrospective Look Two to Four Years Postoperatively	J Bone Joint Surg Am	120	15
4	Crawford CH, et al. [13]	2010	Growth Modulation by Means of Anterior Tethering Resulting in Progressive Correction of Juvenile Idiopathic Scoliosis A Case Report	J Bone Joint Surg Am	119	7.44
5	Stokes IAF [16]	2007	Analysis and simulation of progressive adolescent scoliosis by biomechanical growth modulation	Eur Spine J	111	5.84
6	Newton PO, et al. [17]	2020	Anterior Spinal Growth Modulation in Skeletally Immature Patients with Idiopathic Scoliosis A Comparison with Posterior Spinal Fusion at 2 to 5 Years Postoperatively	J Bone Joint Surg Am	108	18
7	Newton PO, et al. [18]	2008	Spinal growth modulation with an anterolateral flexible tether in an immature bovine model	Spine (Phila Pa 1976)	91	5.06
8	Betz RR, et al. [19]	2010	Vertebral Body Stapling A Fusionless Treatment Option for a Growing Child With Moderate Idiopathic Scoliosis	Spine (Phila Pa 1976)	90	5.63
9	Newton PO, et al. [20]	2008	Spinal Growth Modulation with Use of a Tether in an Immature Porcine Model	J Bone Joint Surg Am	75	4.17
10	Braun JT, et al. [21]	2006	Mechanical modulation of vertebral growth in the fusionless treatment of progressive scoliosis in an experimental model	Spine (Phila Pa 1976)	66	3.3

The most influential paper, authored by Samdani et al., reported the two-year outcomes of anterior vertebral body tethering (VBT) for idiopathic scoliosis. This retrospective study demonstrated that anterior VBT is a safe procedure capable of achieving progressive correction. The first paper on VBT ranks fourth on the list.

**Table 3:** Top 10 most productive authors for minimally invasive scoliosis surgery in adolescent idiopathic scoliosis.

Rank	Author	Affiliations	Country	Records	Main Author	Co-Author	Citations	H-Index
1	Parent S	University of Montreal	Canada	32	3	29	647	15
2	Aubin CE	Polytechnique Montréal	Canada	18	13	5	330	12
3	Samdani AF	Shriners Hospitals for Children	USA	16	5	11	540	9
4	Newton PO	Rady Children’s Hospital	USA	15	12	3	637	11
5	Betz RR	Institute for Spine and Scoliosis	USA	14	3	11	633	11
6	Baroncini A	RWTH Aachen University Clinic	Germany	13	10	3	188	10
7	Trobisch PD	Eifelklinik St. Brigida	Germany	12	7	5	160	8
8	Chang DG	Inje University Sanggye Paik Hospital	South Korea	11	6	5	61	5
	Larson AN	Mayo Clinic	USA	11	7	4	115	5
	Villemure I	Polytechnique Montréal	Canada	11	6	5	398	9
	Castelein RM	University Medical Centre Utrecht	Netherlands	11	5	6	437	7
9	Kim HJ	Inje University Sanggye Paik Hospital	South Korea	10	5	5	40	4
	Pahys JM	Shriners Hospitals for Children-Philadelphia	USA	10	2	8	404	6
	Qiu Y	The Affiliated Drum Tower Hospital	China	10	5	5	129	6
10	Braun JT	University of Utah	USA	9	8	1	372	8
	Grewal H	Cooper Medical School at Rowan University	USA	9	1	8	475	7
	Milbrandt TA	Mayo Clinic	USA	9	0	9	87	4
	Suh SW	Korea University Guro Hospital	South Korea	9	3	6	59	5
	Yang JH	Korea University Anam Hospital	South Korea	9	7	2	59	5
	Zhu ZZ	Nanjing Drum Tower Hospital	China	9	1	8	117	6

## Data Availability

The data presented in this study are available on request from the corresponding author due to privacy restrictions.

## Abbreviations

The following abbreviations are used in this manuscript:

MISS	Minimally invasive scoliosis surgery
AIS	Adolescent idiopathic scoliosis
VBT	Vertebral body tethering
PSI	Pedicle screw instrumentation
RD	Rod derotation
COSS	Conventional open scoliosis surgery
WOS	Web of Science
TLS	Total link strength
